# A unique case of inflammatory fibroid polyp in the duodenum of a female adolescent: Case report and literature review

**DOI:** 10.1097/MD.0000000000006131

**Published:** 2017-02-24

**Authors:** Mauricio Giusti Calderon, Valéria Campos Caivano, Sauro Bagnaresi, José Ozório de Oliveira Lira, Rodrigo Daminello Raimundo, Luiz Carlos de Abreu, João Antonio Correa

**Affiliations:** a Medical Assistant, Pediatric Surgery Department; b Medical Chief of Pediatric Surgery, Hospital do Servidor Público Municipal de São Paulo (HSPM), São Paulo; c Laboratório de Delineamento de Estudos e Escrita Científica, Faculdade de Medicina do ABC (FMABC), Santo André, Brazil; d Full professor, Angiology and Vascular Surgery, Faculdade de Medicina do ABC (FMABC), Santo André, Brazil.

**Keywords:** case report, children, duodenum, inflammatory fibroid polyp, literature review, vanek's tumor

## Abstract

**Background:**

Inflammatory fibroid polyp (IFP) is a very rare benign condition in children that can occur throughout the gastrointestinal tract. It is characterized as a polypoid lesion originating in the submucosa, composed of connective tissue and eosinophilic infiltrate. It is most common in the stomach and in adults between the fifth and seventh decades of life. Its occurrence is unusual in the duodenum.

**Case Summary:**

One case of duodenal IFP was described and the literature is reviewed with emphasis on the clinical and pathological features of IFP in children. A case of an IFP in the duodenum of a 13-year-old girl, who presented with abdominal pain, weight loss, vomiting, and constipation. The patient underwent exploratory laparotomy; a stenosing tumor of the third duodenal portion was found. The affected segment was resected and an end-to-end anastomosis between the duodenum and jejunum segment was performed. Immunohistochemically, actin and CD34 were positive, Ki67 was positive in <1% of cells, and the proteins CD117 and S100 were negative.

**Conclusion:**

To our best of our knowledge, this is the fourth report of IFP in adolescents, the first in a female's duodenum.

## 1 Introduction

First described by Vanek in 1949,^[1]^ who showed that 6 cases had a peculiar lesion of the stomach, described as “gastric submucosal granuloma with eosinophilic infiltration” the denomination inflammatory fibroid polyp (IFP) was coined by Helwig and Ranier in 1953.^[2]^

It occurs mainly in adults between the fifth and seventh decades of life; being rare in children, affects both sexes and can appear in all ages groups.^[3]^ It affects the stomach (66%–75%), followed by the small intestine (18%–23%), mainly ileum, colon, and rectum (4%–7%), gallbladder (1%), esophagus (1%), duodenum (1%), and appendix (<1%).^[3,4]^ Since instances, synchronic and metachronic disease has already been described.^[3]^

Its pathogenesis is still unclear; it is associated with a situation similar to benign reactive response of a granuloma, which occurs in response to an unknown irritating stimulus, which may be trauma, allergic reaction, genetic tendency, bacterial, parasitic infestations, physical, chemical, or metabolic stimulus.^[3–5]^

The IFP is a benign disease of the gastrointestinal tract, described as a polypoid lesions usually localized in the gastric submucosa, nonencapsulated, microscopically composed of loose connective tissue, benign proliferation of spindle-shaped cells, blood vessels, and inflammatory response with infiltrate predominantly eosinophilic, were often arranged in perivascular or periglandular fashion, forming an “onion-skin” appearance.^[3,4,6–8]^

Based solely on their histology, Navas-Palacios et al^[9]^ suggested that it is a true neoplasm that had both vascular and neural origin. However, immunohistochemical studies contradict this possibility since the enolase neuron-specific protein S100 and the antigen-factor VIII in nonproliferating cells were not found.^[3,10]^ Already vimentin and CD34 markers were found in 100% of analysis, and the presence of this last suggests a possible development from primitive vascular or perivascular cells.^[11]^ The presence of strongly positive CD35, overexpressed cyclin D1, fascin, and calponin, in Pantanowitz et al's study, suggests that the stromal cells are of dendritic cell origin with possible myofibroblastic differentiation.^[5]^ The possibility of IFP be a subtype of gastrointestinal stromal tumor (GIST) is discarded since the CD117 (c-kit) marker is not found.^[5,12]^

Clinical presentation depends on the site of involvement.^[13]^ The duodenum IFP is more associated to nonspecific symptoms like weight loss, colicky abdominal pain, or dyspeptic symptoms, with or without signs and symptoms of obstruction or bleeding from the upper gastrointestinal tract.^[12]^

## 2 Case report

### 2.1 Clinical presentation

A 13-year-old female, white patient, born in São Paulo, presented to our emergency clinic complaining of abdominal cramping-type pain for 7 months, associated with nausea and vomiting, not related to supply and relieve abdominal discomfort, constipation, and weight loss of + −3 kg in the period. She denied fever, gastrointestinal bleeding, and hematuria. Diagnostic investigation was performed with laboratory tests that showed no significant changes; upper gastrointestinal endoscopy and computed tomography (CT) abdomen showed mild erosive esophagitis with a marked gastric distension and gastroduodenal stasis on 2^nd^ and 3^rd^ portions, and with altered 3^rd^ and 4^th^ duodenal portions, suggestive of extrinsic compression respectively. After these results, she was treated with domperidone, omeprazole, and scopolamine, being referred to outpatient treatment.

**Figure 1:**
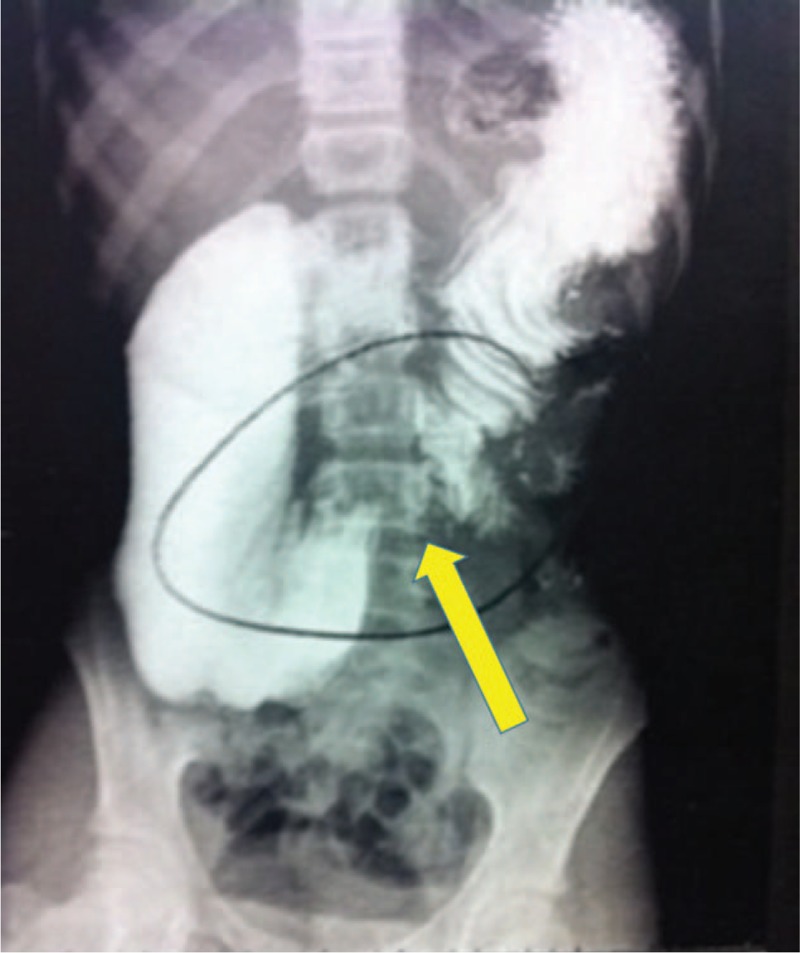
Contrasted abdominal x-ray showing duodenal dilatation with a progression stop of the contrast in the transition between 3^rd^ and 4^th^ duodenal portions.

She was returned once more to our emergency clinic with the same complaints and was hospitalized for investigation. A contrast x-ray was performed that showed a large duodenal dilatation with a progression stop of the contrast in the transition between 3^rd^ and 4^th^ duodenal portions (Fig. 1).

**Figure 2:**
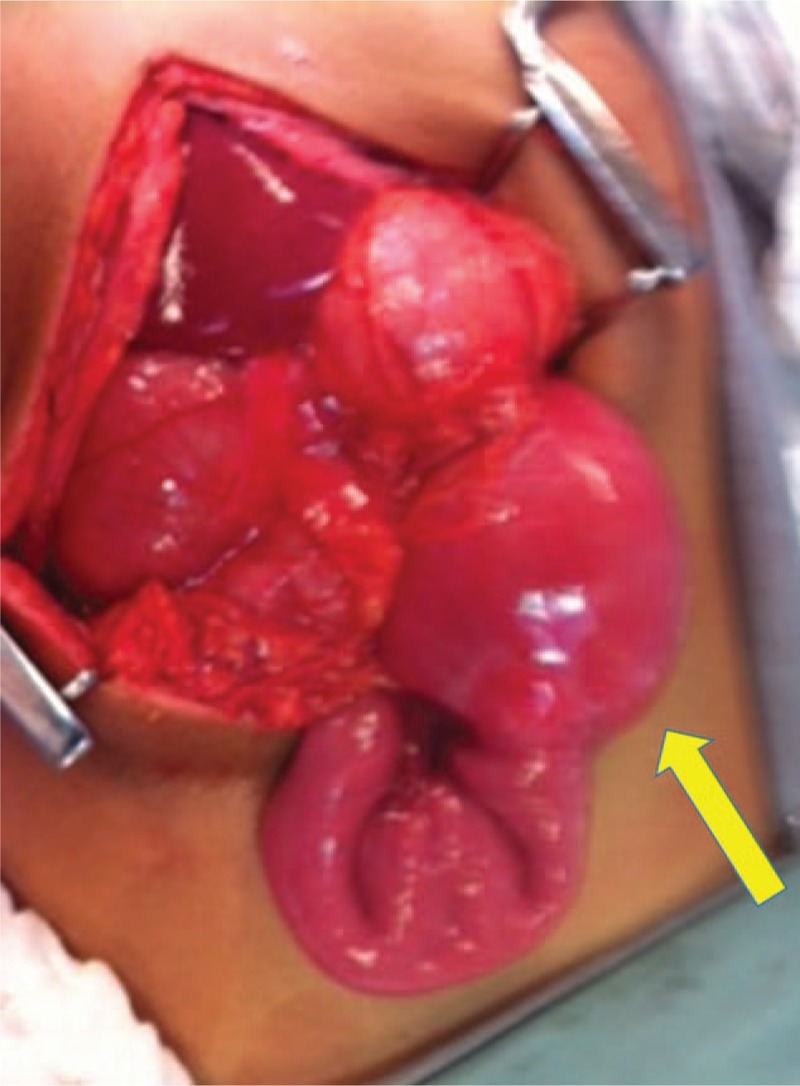
3^rd^ duodenal portion appears dilated where the tumor was located. Intraoperative photo.

**Figure 3:**
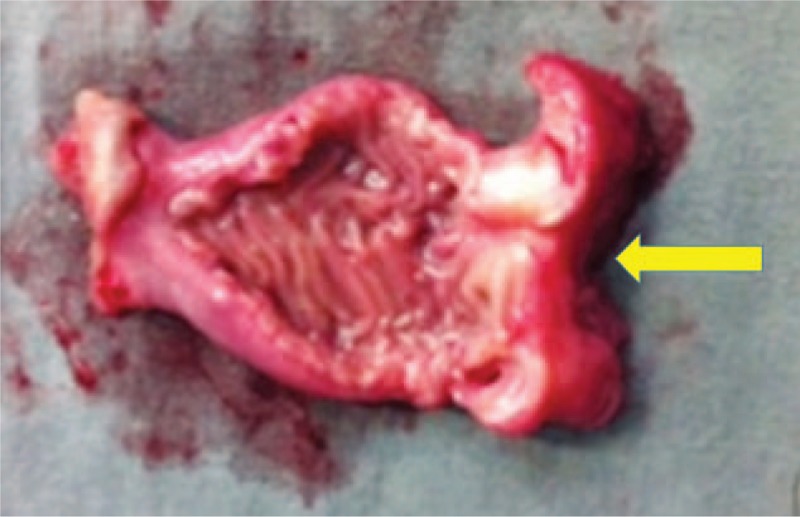
Gross image of opened duodenum containing the tumor.

The patient underwent exploratory laparotomy, in which stenosing tumor was found of the third duodenal portion (Fig. 2), intramural, of 40 × 25 × 10 mm (Fig. 3). A resection of the affected segment and restoration of intestinal transit with end-to-end anastomosis between the duodenum and jejunum segment were performed. Patient had a good postoperative course and was discharged on the 14th postoperative day.

## 3 Materials and methods

This study has ethics committee aproval of Faculdade de Juazeiro do Norte: CAAE: 56635016.5.0000.5624 (Plataforma Brasil).

### 3.1 Pathologic evaluation

**Figure 4:**
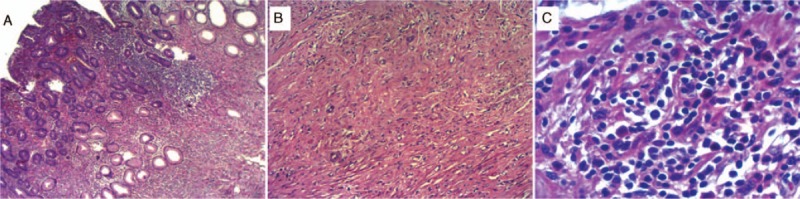
(A) At low magnification, presence of submucosal tumor with bland spindle cells, collagenous stroma, and eosinophilic inflammatory infiltrates. (B) Spindle cells proliferation with vascular neoformation. (C) High magnification, classical presence of eosinophilic inflammatory infiltrates.

Histologic sections from tissue samples of the duodenal tumor had been processed routinely in 10% buffered formalin and embedded in paraffin, that were stained with hematoxylin and eosin (H&E) for confirmation of the diagnosis (Fig. 4A–C).

### 3.2 Immunohistochemical evaluation

It was performed on paraffin-embedded tissue by standard techniques using actin/HHF-35 (DBS; 0.5:1500 dilution), CD34 (Dako; 1:200 dilution), CD117/C-kit (Cell Marque; 1:40 dilution), Ki67 (Cell Marque; 1:250 dilution), and S-100 (Dako, 0.5:2000 dilution) stains.

## 4 Results

**Figure 5:**
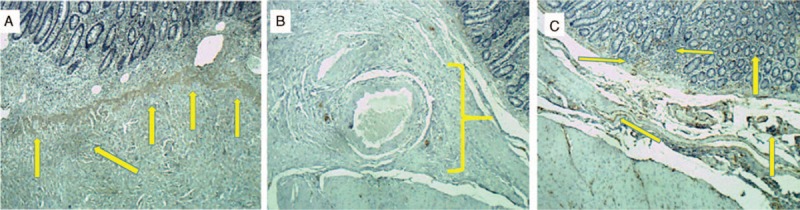
Actin immunohistochemical stains. (A) Demonstrating strong actin immunoreactivity. (B) Enlargement of duodenal submucosa. (C) Mucosa and submucosa demonstrating strong actin immunoreactivity.

**Figure 6:**
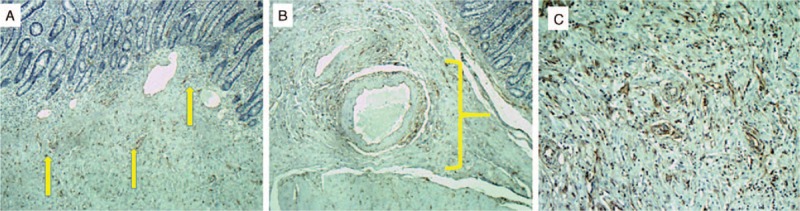
CD34 immunohistochemical stains. (A) Demonstrating weak CD34 immunoreactivity. (B) Enlargement of duodenal submucosa. (C) High magnification.

Pathological examination of the tumor showed a stenosing white lesion with 40 × 25 × 10 mm. At the submucosa and muscular layer, a hyperplastic process characterized by proliferation of spindle cells resembling fibroblasts, newly formed capillaries and abundant eosinophils. Immunohistochemically, actin **(**Fig. 5A–C**)** and CD34 **(**Fig. 6A–C**)** were positive, Ki67 was positive in less than 1% of the cells, as the protein CD117 and S100 were negative.

## 5 Discussion

The literature on the IFP in children and adolescents is limited to case reports and case series as shown in the table below.

As seen in Table 1, only 18 pediatric and 3 adolescent cases of IFP have been published in medical literature until 2015. Adding our case to this series, we have an age range of 2 to 15 years (mean 6.85 years), predominantly male patients (64%), and a higher incidence of small bowel involvement (53%), followed by the stomach (13.5%), colon (13.5%), duodenum (13.5%), and rectum (6.5%). This statistic, possibly by limited number of cases, is quite different from that found in adults, in which the peak incidence is between the fifth and seventh decades of life; it equally affects both sexes and the stomach is most affected organ.^[2–4,8]^

**Table 1:**
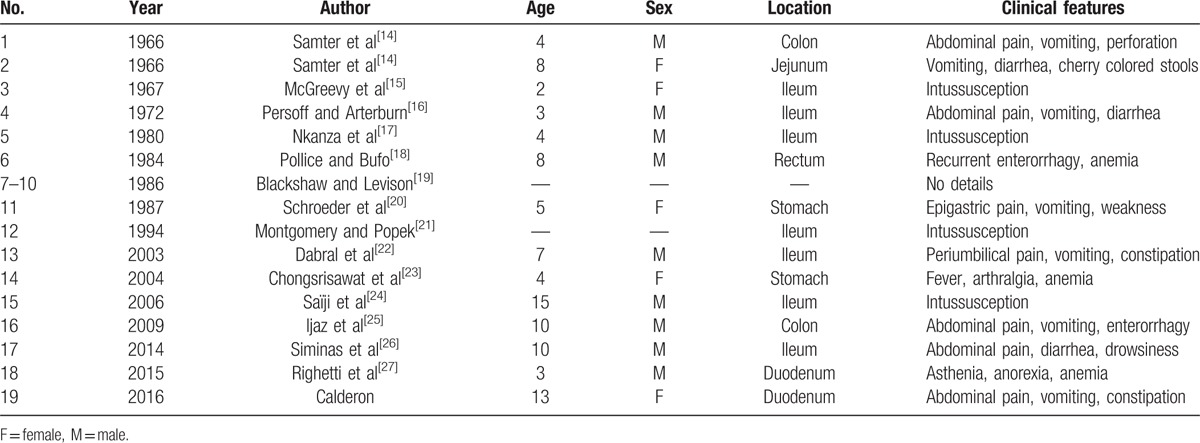
Literature review on inflammatory fibroid polyp in children.

The symptoms depend on the anatomical location of the polyp; in adults, at the duodenum, the most common symptoms are abdominal pain and bleeding from the upper gastrointestinal tract,^[3]^ but in children, the most frequent symptoms were, abdominal pain, vomiting, diarrhea, asthenia, and anemia. Righetti et al described the first case of IFP in a 3-year-old child, in whom symptoms were asthenia and anorexia, with no alterations on physical examination; they performed a partial en block duodenectomy and an inverted Y plasty duodenal repair.^[27]^

Additional tests may be used in diagnostic research; however, it is rare to perform the correct preoperative diagnosis of IFP. Several tests are used to reach an accurate diagnosis, x-rays, ultrasound, CT scan, magnetic resonance imaging, upper gastrointestinal endoscopy, and endoscopic ultrasound (EUS), but is after laparotomy that usually the diagnosis is made.^[3,4,6,8,9,20,23]^

Endoscopy can be used for duodenal IFP, as for esophageal and gastric. Usually shown as intraluminal, protruding, solitary polypoid or sessile, intramural lesion, with a smooth and often ulcerated mucosa.^[3,19,28]^ In EUS, lesions show up with indistinct margins, are hypoechogenic and homogeneous, and are located within the second and third sonographic layers of the gastric wall^[3]^; nevertheless, at the children's duodenum, in Righetti et al’ study, EUS showed an hypoechoic mass located into the submucosa (echo-layer 3), not completely separated from the muscularis propria (echo-layer 4).^[27]^

The treatment of choice is complete resection of the IFP. Endoscopic polypectomy is the technique of choice when there is a polypoid lesion of up to 2 cm, usually from the stomach or colon. Once the IFPs have a submucosal origin and are sessile, endoscopic resection can result in incomplete resection or perforation, increasing the chance of local recurrence.^[3,23,27,29]^

As seen in our case, IFP is histologically characterized by a submucosal lesion composed of proliferating fibroblasts and blood vessels, accompanied by eosinophilic infiltration.^[23]^ Involvement of the mucosal layer is very frequent, 89% to 93.5%.^[7,13]^ The presence of concentric cuffing of vessels by the lesional cells, referred to as an “onion skin” appearance, was not found in our study. Liu et al^[13]^ found this characteristic perivascular onion skin feature in only 54% of cases.

Immunohistochemical profile revealed the presence of actin and CD34; the absence of CD117 and S100 is compatible with other author's findings. In addition, the absence of CD117 (c-kit) is important for the differentiation between IFP and GIST.^[3–5,9–11,13,29]^

## 6 Conclusion

The IFP is a rare disease with only 18 pediatric cases described in the literature. Its pathogenesis is still unknown; the symptoms depend on their location. The preoperative diagnosis is rarely established before the operation and is usually made after resection. To the best of our knowledge, this is the fourth report of IFP in adolescents, the first in a female's duodenum.

## Acknowledgments

The authors are grateful to Dr. Sérgio R R Araújo, pathologist, for providing the microscopic images, and to the members of the Scientific Writing Laboratory, of Faculdade de Medicina do ABC (FMABC), by the critical analysis.
